# Total Iodine Quantification and In Vitro Bioavailability Study in Abalone (*Haliotis discus hannai*) Using Inductively Coupled Plasma Mass Spectrometry

**DOI:** 10.3390/foods13091400

**Published:** 2024-05-02

**Authors:** Hansol Doh, Min Hyeock Lee

**Affiliations:** 1 Department of Food Science and Biotechnology, Ewha Womans University, 52 Ewhayeodae-gil, Seodaemun-gu, Seoul 03760, Republic of Korea; hdoh@ewha.ac.kr; 2 Department of Biotechnology, College of Life Sciences and Biotechnology, Korea University, 145 Anam-ro, Seongbuk-gu, Seoul 02841, Republic of Korea

**Keywords:** abalone (*Haliotis discus hannai*), iodine, in vitro bioavailability, digestion efficiency, absorption efficiency

## Abstract

The aim of this study is to determine the total iodine content in Korean abalone (*Haliotis discus hannai*) and to investigate the bioavailability of iodine using an in vitro method. This research paper focuses on total iodine quantification in abalone (*Haliotis discus hannai*) and its components (viscera and muscle) using inductively coupled plasma mass spectrometry (ICP-MS). Additionally, an in vitro bioavailability study explored iodine absorption potential. Abalone pretreatment involved both the European standard method (ES) and microwave-assisted extraction method (MAE). The limits of detection (LOD) were 0.11 ng/g for both ES and MAE, with a limit of quantification (LOQ) of 5.4 ng/g for MAE. Accuracy, assessed using a reference material (fish muscle, ERM—BB422), showed values of 1.5 ± 0.010 mg/kg for ES and 1.6 ± 0.066 mg/kg for MAE, within an acceptable range of 1.4 ± 0.42 mg/kg. Precision, evaluated using the Horwitz ratio (HorRat) with a reference material, was determined to be 0.45 for ES and 0.27 for MAE. Therefore, total iodine contents were estimated as 74 ± 2.2 µg/g for abalone viscera and 17 ± 0.77 µg/g for abalone muscle with ES, and 76 ± 1.0 µg/g for abalone viscera and 17 ± 0.51 µg/g for abalone muscle with MAE. Recovery tests demonstrated an acceptable range of 90–110%. In the in vitro bioavailability assessment, digestion efficiency yielded ranges of 42–50.2% for viscera and 67–115% for muscle. Absorption efficiency variations were determined as 37–43% for viscera and 48–75% for muscle.

## 1. Introduction

*Haliotis discus hannai*, also known as abalone, is renowned for its health benefits in East Asia. Although abalone is primarily produced and consumed in East Asia, there is a possibility of increased consumption in Western countries due to the current interest in health functional foods. Abalone is rich in various proteins and minerals such as calcium, phosphate, vitamin B_1_, and B_2_ [1]. Additionally, owing to taurine, abalone aids in the protection of the liver and intestines, fatigue recovery, and prevention of cardiac infarction [2]. Therefore, with the growing interest in abalone, its production has increased dramatically, with S. Korea and China being the main contributors [3]. Among all the health functional substances in abalone, this study specifically focuses on iodine because iodine is one of the most important trace elements in our body for synthesizing thyroid hormones [4]. Oceanic organisms accumulate a significant amount of iodine through bioaccumulation from seawater [5]. Similarly, other studies also discuss bioaccumulation among marine organisms [6,7,8]. The findings from previous studies, indicating that abalone primarily feed on seaweeds such as kombu and wakame, make it reasonable to assume that abalone contains a substantial amount of iodine [1,2].

Iodine is a primary component of thyroid hormones, crucial for managing various metabolic processes in our body, including growth, brain development, muscle function, heart health, pituitary function, and kidney function. Particularly, iodine deficiency can lead to irreversible brain damage in infants and children [9,10]. Consequently, iodine deficiency disorders (IDD) continue to be a longstanding global issue [11,12]. According to previous studies [9,13,14], approximately 30% of the world′s population still suffers from IDD. This is the case not only in Central Asia, where it is challenging to supply iodine-rich products, but also in Europe, where 44%, including school-age children in the United Kingdom, still experience insufficient iodine intake. The United States and Australia face similar problems [15]. Various research efforts have been undertaken to address this issue, including the production of iodine-fortified foods such as salts [9], iodine edible films [12], iodine microcapsules [16], and the study of iodine content in marine products [17,18]. However, excessive iodine intake can also pose serious health risks, leading to conditions such as goiter, hyperthyroidism, and hypothyroidism [19,20]. Therefore, the accurate determination of iodine levels is a critically important issue.

Three steps should precede the analysis of iodine content in abalone. Firstly, for precise iodine analysis, selecting an appropriate solvent is crucial. Some research articles use acidic solvents to decompose the sample matrix for iodine determination [21], but there is a possibility of sublimation due to iodine′s high volatility [22]. Tetramethylammonium hydroxide (TMAH) has been used in numerous studies for exact iodine determination as alkaline solvents maintain iodine stability [18,23]. Although some alkaline solvents, like ammonia media, may affect accuracy by inducing instrumental or memory effects in inductively coupled plasma mass spectrometry (ICP-MS), this study opts for TMAH [24]. Despite controversies regarding TMAH as a proper solvent for iodine analysis, this study chose it based on there being no issues observed in preliminary experiments.

Secondly, researchers should select the correct pretreatment method for decomposing the sample matrix. Alkali ashing [25], microwave-assisted extraction (MAE) [18], the European standard method [26], and microwave-induced combustion [17] have been investigated. In this study, the European standard method (ES) and MAE were chosen and compared for their simplicity and precision among all methods. Additionally, by comparing these two certified methods and through their respective validation tests, an accurate iodine value was obtained.

Thirdly, various methods have been employed to determine total iodine content [27], such as the reduction–oxidation reaction [28], neutron activation analysis [29], X-ray fluorescence spectrometry [30], ion chromatography [31], high-performance liquid chromatography [32,33], inductively coupled plasma optical emission spectrometry (ICP-OES) [34,35], and ICP-MS [23,25,26,36]. Among all these determination methods, this study used ICP-MS due to its superior sensitivity for analyzing total iodine content, characterized by a low limit of detection and high precision, compared to other methods [27].

In food nutrition studies, bioavailability is a crucial factor as it encompasses the entire process from ingestion to elimination. It provides insights into how specific nutritious elements are absorbed in our bodies. Building upon previous research modifications [37], this study successfully estimated the potential iodine intake from abalone by adjusting pH, utilizing gastric/intestinal juice with enzymes, and employing a practical dialysis membrane. This method offers a simple, rapid, and cost-effective approach to estimating iodine in the in vitro bioavailability study of abalone.

Therefore, the objective of this study is to determine the exact total iodine content in abalone and assess its bioavailability using an in vitro method.

## 2. Materials and Methods

### 2.1. Reagents and Equipment

Ultrapure water with a resistance of 18.2 MΩ·cm was prepared using a Milli-Q water purification system (Millipore, Billerica, MA, USA). All reagent additions and dilutions were conducted using this ultrapure water. Potassium iodide (Duksan Pure Chemical Co., Ltd., Ansan, Republic of Korea) served as the standard material, and a tellurium solution (Kanto Chemical Co., Ltd., Tokyo, Japan) was used as an internal standard for iodine. TMAH (25% in water) (Tokyo Chemical Industry Co., Ltd., Tokyo, Japan) was prepared for sample pretreatment. Nitric acid and hydrochloric acid (33–36%) were obtained from Duksan Pure Chemical Co., Ltd. (Ansan, Republic of Korea). Pure-grade sodium hydroxide was sourced from Samchun Pure Chemical Co., Ltd. (Pyeongtaek, Republic of Korea). Pepsin from porcine gastric mucosa (≥250 units/mg solid) and bile extract salts were purchased from Sigma-Aldrich (St. Louis, MO, USA). Pancreatine from porcine pancreas was obtained from Wako Pure Chemical Industries Ltd. (Osaka, Japan). Additionally, 99% piperazine-1,4-bis (2-ethane-sulfonic acid) disodium salt (PIPES) was acquired from Tokyo Chemical Industry Co., Ltd. (Tokyo, Japan). The reference material (Fish muscle; ERM—BB422, European Commission, Joint Research Centre, Institute for Reference Materials and Measurements, Geel, Belgium) was used to verify the accuracy in determining iodine contents. The tuning solution for ICP-MS 7500cs was obtained from Agilent Technologies (Palo Alto, CA, USA).

A dry oven (FO-600M, Jeio Tech Co., Ltd., Daejeon, Republic of Korea) was used for the ES method and for eliminating water traces. The MAE method was performed using a microwave (MILESTONE S&T Co., Ltd., Seoul, Republic of Korea), and a Teflon microwave vessel was used. Iodine content was analyzed by ICP-MS (Agilent Technologies, Palo Alto, CA, USA) equipped with an Agilent I-AS auto-sampler. The sample introduction mode was PeriPump, and the nebulizer was in Micro Mist mode. The collision/reaction cell was operated in quadrupole mode, and its detector was equipped with a G3292A recirculating chiller from Agilent Technologies (Palo Alto, CA, USA). Additional details are provided in Table 1. For the in vitro bioavailability test, a water bath (BS-10 from Jeio Tech Co., Ltd., Daejeon, Republic of Korea) was used. Spectra/Por^®^ Membrane Dialysis RC Tubing (2 kDa molecular weight cut-off dialysis membrane; 10 m length, dry diameter 24 mm, and 4.6 mL/cm) was obtained from Spectrum Laboratories, Inc. (Rancho Dominguez, CA, USA). An ORION STAR A211 pH-meter (Thermo Scientific, Waltham, MA, USA) with a plastic electrode was used for pH measurements. A centrifuge from Hanil Science Industrial Co., Ltd. (Seoul, Republic of Korea) was also utilized. To prevent possible contamination from the environment, 10% nitric acid was used to clean glassware over a 2-day period. After cleaning with 10% nitric acid, glassware was washed three times with ultrapure water.

### 2.2. Samples

**Figure 1:**
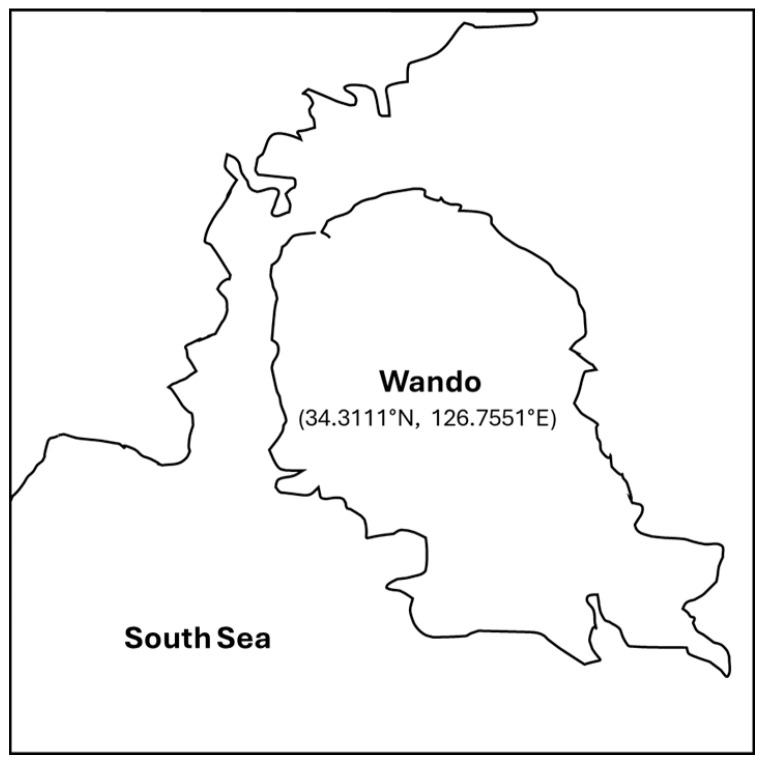
Representation of the Wando, the location where the abalone (Haliotis discus hannai) samples were collected.

Abalone (*Haliotis discus hannai*) from Wando, located in the southwestern region of S. Korea, was generously donated by the Departments of Marine Product Cultivation (Wando-gun office, Wando, South Jeolla province, Republic of Korea) (Figure 1). The target samples were abalone viscera (AV) and abalone muscle (AM). Initially, to obtain samples, the abalone shell was separated from the body. Subsequently, AV were separated from AM and freeze-dried. After freeze-drying, the samples were pulverized using a commercial grinder for 15 min. All ground samples were stored in 50 mL PP tubes. Three AV and AM samples were randomly selected and mixed together in separate 50 mL PP tubes. Before pretreatment, the samples were placed in a 40 °C dry oven to eliminate trace water. The reference material used was fish muscle. Blanks were prepared in four ways: calibration blank, reagent blank, method blank, and drift correction blank. All blanks were prepared in 1.0% TMAH and included the internal standard. The results were expressed as a dry basis.

### 2.3. Sample Pretreatment Methods

Two pretreatment methods were compared in this study to obtain more accurate data. The first method is based on the ES with slight modifications [26,38]. A 0.20 g sample was placed into a 50 mL PP tube. Then, 5.0 mL of ultrapure water and 1.0 mL of 25% TMAH solution were added. The mixture was kept at 90 °C in a dry oven for 3 h. Subsequently, the samples were diluted with ultrapure water, centrifuged at 4000 rpm for 15 min, and filtered with a 4.5 µm filter using a 10 mL syringe. Once the supernatant was separated, the residues were carefully washed with small amounts of ultrapure water. After the 4.5 µm filter, the supernatant was added to a new 50 mL PP tube. The final volume was set as 3 mL for further experiments.

Secondly, MAE, which has been widely used for iodine analysis in many studies, was adopted [17,18,39]. A 0.2 g sample was placed into a Teflon microwave vessel. Then, 5 mL of ultrapure water and 5 mL of 25% TMAH were added. After closing the vessel cap, the microwave system was operated. The microwave system consisted of a 10 min ramp time from ambient temperature to 200 °C. Vessels were kept for 5 min at 200 °C, and a cooling step was performed to reach 25 °C. Then, the samples were transferred to a 50 mL PP tube and centrifuged at 4000 rpm for 15 min. Supernatants were collected, and residues were rinsed with ultrapure water and centrifuged again under the same conditions. After the 4.5 µm filter using a 10 mL syringe, the supernatant was added to a new 50 mL PP tube. The final volume was set as 3 mL for further experiments.

### 2.4. Validation Methods

All validation methods adhered to the AOAC and Codex guidelines [40,41]. To ensure precision for the equipment, four types of blanks were utilized: the calibration blank, reagent blank, method blank, and drift correction blank [24]. The drift correction blank was composed of a 5 µg/L standard solution to check for system/memory effects and any possible contamination of the ICP-MS. Drift correction blanks were allocated at intervals of 3 to 6 for each experiment. The 5 µg/L standard solutions were estimated and checked to ensure they ranged from 80% to 120% [42]. *LOD* and *LOQ* were calculated with the method blank as follows [41]:

(1) LOD=0.4×ML

(2) $$LOQ=Average\left(blank\right)+10\times SD$$

where, *ML* is the minimum detected amount of iodine, and *SD* is standard deviation of the blanks.

The accuracy of the test was assessed using the reference material (fish muscle, ERM—BB422, with an acceptable range of 1.4 ± 0.42 mg/kg) and the precision of the test was determined with the Horwitz ratio (HorRat) (calculated by RSD_r_/pRSD_R_, where RSD_r_ indicates the relative standard deviation of repeatability and pRSD_R_ means predicted relative standard deviation of reproducibility). The study was verified through the analysis of 7 reference material samples.

### 2.5. Iodine Determination

Following the specified conditions (Section 2.1), iodine contents were determined. Samples consisted of calibration blanks, reagent blanks, method blanks, drift correction blanks, AV, and AM. Prior to measurements, all samples were diluted at ratios ranging from 1:2 to 1:20, and 100 µL of tellurium (^125^Te) solution (2 mg/L) was added to each sample as an internal standard for iodine analysis. Experiments were conducted in triplicate.

### 2.6. Recovery Test

A recovery test using spike solutions was also conducted. Low (5 µg/L) and high (10 µg/L) spike solutions were prepared by diluting standard solutions and subsequently added to each sample for analysis by ICP-MS. The reference material was applied in this study, and recovery calculations were performed for both AV and AM, using the total iodine determination method as described in Section 2.5. The internal standard (^125^Te) was also added to ensure accuracy. All experiments were carried out in triplicate. The acceptable range followed the AOAC official methods of analysis guideline [40].

### 2.7. In Vitro Bioavailability Study Procedure

The in vitro bioavailability study was conducted with a slight modification of a previous study [43]. Initially, 0.5 g samples (reference material, AV, and AM) were placed into 100 mL Erlenmeyer flasks. Subsequently, 20 mL of ultrapure water was added. The mixture was left to stand for 15 min, and then the pH was adjusted to 2.0 using 6.0 M hydrochloric acid. Concurrently, 0.15 g of gastric solution (6%, *w*/*v*, pepsin dissolved in 0.1 M hydrochloric acid) was introduced. The flasks were covered with aluminum foil and incubated in a water bath conducive to horizontal vibration. Maintaining conditions at 37 °C and 150 rpm, the flasks were incubated for 2 h. Following incubation, the flasks were transferred to an ice bath to halt enzyme activity. Subsequently, 5 mL of intestinal solution (4%, *w*/*v*, pancreatin and 2.5%, *w*/*v*, bile salt dissolved in 0.1 M sodium bicarbonate) was added to the flasks. At this juncture, a 2 kDa dialysis membrane, filled with 20 mL of 0.15 N PIPES solution (pH 7.5 adjusted with 0.1 M hydrochloric acid; 12 cm long), was placed into the Erlenmeyer flasks. Following conditions of 37 °C and 150 rpm for 2 h, the external surface of the dialysis membrane was gently cleaned with ultrapure water. The solution inside the membrane (dialysate) and the residual fractions outside were both transferred to 50 mL PP tubes and stored at −20 °C until measurement. For pH determination, thawed separated samples were naturally brought to ambient temperature and their pH was measured using a pH meter. To analyze iodine content after the in vitro bioavailability study procedure, the dialysate passed through a 0.45 µm membrane filter, and 5 g of the external residual fractions (in slurry form) underwent MAE once again under the same conditions as described in Section 2.3. Subsequently, the iodine contents of the dialysate and external residual fractions were combined for the mass balance study. A method blank was employed to calculate the limit of determination. Digestion and absorption efficiencies were calculated as follows:

(3) $$Digestion\;efficiency(\%)=\frac{Iodine\;contents\;in\;inner\;and\;outer\;membrane\;}{Total\;iodine\;contents\;of\;samples}\times 100$$

(4) $$Absorption\;efficiency(\%)=\frac{Iodine\;content\;in\;inner\;membrane}{Iodine\;contents\;in\;inner\;and\;outer\;membrane}\times 100$$

### 2.8. Statistical Analysis

All data are presented as the mean ± standard deviation. The data were analyzed using the independent samples *t*-test. The Statistical Package for Social Sciences software (SPSS, Version 20.0, SPSS Inc., Chicago, IL, USA) was used to calculate a *t*-value and *p*-value for the *t*-test. Tests were implemented in order to check for a significant difference relation between two groups. The significance level was *p* < 0.05.

## 3. Results and Discussion

### 3.1. Optimization of ICP-MS Condition

In the previous study, attention was primarily focused on the auto-sampler and the areas associated with uptake tubing and nebulizing, and the spray chamber to identify potential contamination and system/memory effect factors [24]. Therefore, choosing a solvent for washing the tubing and nebulizing areas and the spray chamber was crucial. In this study, a washing solution comprising 1.0% TMAH and ultrapure water was selected as it contributed to stabilizing the intensity of ICP-MS for iodine content analysis (ICP-MS intensity expressed as counts per second, CPS). The acceptable range for the CPS of ICP-MS was verified using an ICP-MS tuning solution. Subsequently, the ICP-MS was operated for 2 h before iodine content analysis, involving 1 h of washing with ultrapure water and another 1 h of cleaning with 1.0% TMAH. During this process, the background CPS dropped sharply and stabilized. The system/memory effect of ICP-MS was consistently monitored with a 5 µg/L drift correction blank to identify any potential system/memory effects or contamination from other factors.

### 3.2. Method Validation

#### 3.2.1. Linearity

For each new calibration standard, iodine concentrations were prepared ranging from 1 to 100 µg/L. The standard curve was established with seven levels: 0, 0.5, 1, 5, 10, 20, and 100 µg/L. As mentioned earlier, standard solutions were prepared daily. The correlation coefficient (R^2^) value was consistently above 0.999, indicating excellent linearity for the analysis of iodine in abalone. Table 2 presents the results of the linearity test.

#### 3.2.2. Limit of Determination

The LOD and LOQ are shown in Table 2. Using the ES method, the LOD was 0.11 ng/g, and the LOQ could not be determined because the concentration of method blanks was estimated to be below 0. On the other hand, with MAE, the LOD was 0.11 ng/g, and the LOQ was 5.4 ng/g. In the case of the LOQ of the ES method, the CPS value was lower than the CPS of 0 ppb. This discrepancy occurred because the composition of the samples mainly comprised ultrapure water compared to the calibration blank. Since the CPS of TMAH is higher than ultrapure water, the results are as shown in Table 2. The results from MAE were slightly higher than those from ES. The limits of determination for both methods were calculated using the method blank because the method blank value was higher than that of the reagent blank. Both methods demonstrated very low LOD and LOQ for analyzing iodine content compared to previous studies, which ranged from 14 to 30 µg/L [36,44,45]. Although the LOD and LOQ in this study were slightly higher than in another study [24], the results were considered suitable for the experiments. Thus, iodine contents of the AV and AM were always quantified in this study when the value was above the LOQ for each method.

#### 3.2.3. Accuracy

Table 2 includes the results of the accuracy test. The certified value of fish muscle was 1.4 ± 0.42 mg/kg (according to ISO/IEC Guide 98-3 [46], in the case of iodine, the coverage factor *k* = 2.57 corresponds to a level of confidence of about 95%). Both ES and MAE fell within the acceptable range; 1.50 ± 0.10 and 1.64 ± 0.066 mg/kg, respectively.

#### 3.2.4. Precision

Table 2 shows the results for each parameter of the samples. The HorRat included the concept of assuming intra- and inter-laboratory differences in the quantification [40]. In the intra-laboratory concept, RSD_r_ was 6.7% for ES and 4.0% for MAE. In the inter-laboratory concept, pRSD_R_ was 15.0% for ES and 14.8% for MAE. According to AOAC guidelines, the expected RSD_r_ percentage is 11%, and the pRSD_R_ percentage is 16%. Thus, all RSD_r_ and pRSD_R_ percentages were within the acceptable range. The HorRat value was 0.45 for ES and 0.27 for MAE. These results were acceptable for the HorRat range (0.25–1.34). Therefore, both pretreatments were reasonable for analyzing iodine in abalone.

### 3.3. Iodine Determination in Abalone

Based on various validation methods, iodine determination in AV and AM was investigated. Table 3 presents the results of iodine contents in abalone. The total iodine in AV using the ES was 74 ± 2.2 µg/g, and for AM, it was 17 ± 0.77 µg/g. The total iodine quantification in AV with MAE was 76 ± 1.0 µg/g, and for AM, it was 17 ± 0.51 µg/g. Both pretreatment methods showed a consistent trend that AV has more iodine than AM. East Asia is a primary global area for abalone production, and abalones are usually farmed, often being fed seaweeds such as kombu and wakame [2,3]. Previous studies on seaweeds using a similar method determined the total iodine contents as follows: 77.3 ± 8.69 µg/g in dulse (*Palmaria palmate*), 43.2 ± 3.73 µg/g in nori (*Porphyra umbilicale* and *Porphyra lineariss*), 65.6 ± 2.11 µg/g in sea lettuce (*Ulva rigida*), 6138 ± 313.7 µg/g in kombu (*Laminaria ocholeuca* and *Laminaria sacharina*), and 305.6 ± 42.39 µg/g in wakame (*Undaria pinnatifida*) [37]. Compared to these results, AV showed higher iodine contents than nori and sea lettuce, similar to dulse, and much lower than kombu and wakame. Therefore, while there are considerable iodine contents in AV, they are not as high as those found in their feeds, kombu and wakame. In the case of AM, the iodine contents were lower than most seaweeds. Although seaweeds are generally believed to be rich in iodine, there have been no studies investigating the relationship between seaweeds and marine products. Another study on iodine contents in shrimp (*Litopenaeus vannamei*) reported iodine contents of 17.8 ± 1.0 µg/g in the whole shrimp and 35.2 ± 0.4 µg/g in the shells (shells + head) [17]. This aligns with the results of this study, as shrimp also have viscera in their head.

The reason for the distribution of iodine in abalone being different is not yet identified. Since East Asia is the primary area for global abalone production [2,3], where abalones are typically farmed and fed seaweed, it was hypothesized that abalones might have high iodine content due to bio-accumulation mechanisms. Many previous studies have demonstrated that seaweed contains a substantial amount of iodine and have explained that seaweed has an outstanding bio-accumulation ability [18,21,28,32]. The results of this study can be interpreted in two ways. The first reason is that the elimination ratio of iodine is higher than the accumulation ratio. The second reason is that not only iodine but also other trace elements can accumulate with great diversity based on factors such as families, genera, and species, and even under similar environmental conditions, geographical origins, and harvesting times [18,47]. The general tendency in the ocean is to arrange iodine contents in the order of frequency as follows: marine plants, marine animals, and seawater. However, further data on other marine organisms is needed for generalization [48].

### 3.4. Recovery

As shown in Table 4, reference material, AV, and AM were spiked with both low (5 µg/L) and high (10 µg/L) concentrations of the standard solution. In all samples, ES ranged from 94 to 104%, while MAE varied from 98 to 106%. All values fell within an acceptable range [40]. High recovery (>94%) was observed in all samples. This indicates that both ES and MAE successfully decomposed the abalone matrix, ensuring that all the samples used in this study provide an accurate measure of the total iodine content in the abalone.

### 3.5. In Vitro Bioavailability Study

#### 3.5.1. Preliminary Studies

Many researchers have used sodium bicarbonate or phosphate-buffered saline (PBS) as a buffer in in vitro bioavailability or release studies. However, some studies have suggested using the PIPES solution as a buffer for in vitro bioavailability studies due to its good buffering capacity [37,49]. A preliminary study compared the use of 7.5 PBS buffer, which resulted in a pH higher than 7.5 in the inner membrane dialysis solution (pH of 9.0 ± 0.01 for AW and 9.2 ± 0.005 for AM). This preliminary study indicated that the PBS buffer could not maintain the desired pH. Similar results were observed with the sodium bicarbonate buffer in a previous study [37,43]. Therefore, a PIPES buffer was used in this study, and the data collected is presented in Table 5, demonstrating suitable pH changes inside the membrane in the range of 7.2 to 7.6. In the case of the residual fraction, pH varied from 6.8 to 7.9, with the residual fraction pH being lower than the dialysate except for the method blank. It is suggested that some acidic compounds from AV and AM during the digestion process may affect the pH.

The validation test of the in vitro bioavailability procedure in this study was checked using the method blank. Table 6 presents the limits of determination (LOD and LOQ) of the in vitro bioavailability tests in this study. The cases were separated into dialysate and residual fraction. As described in Section 2.4, the R^2^ value of the calibration curve was consistently over 0.999. Additionally, drift correction blanks (5 µg/L) were allocated at intervals. The LOD and LOQ were estimated with the method blank and calculated as described in Section 2.4. The LOD of the dialysate was 0.87 mg/kg, and the LOQ of the dialysate was 14.04 mg/kg. In contrast, the LOD of the residual fraction was 0.21 mg/kg, and the LOQ was 30.54 mg/kg. These values were higher than the previous LOD and LOQ, as other factors such as enzymes or the PIPES buffer could have affected the results.

#### 3.5.2. Mass Balance Study

To evaluate accuracy, a mass balance study was conducted using AV and AM. In the procedure of the in vitro bioavailability study, the iodine contents in the dialysate and residual fraction were determined. The total determined iodine contents of AV and AM were obtained from Section 3.3. Iodine contents of the dialysate and residual fraction are shown in Table 7. According to the statistical results, there was a significant difference between the total iodine contents and the sum of dialysate and residual fraction of AV in both pretreatment methods. On the other hand, there was no significant difference between the total iodine contents and the sum of dialysate and residual fraction of AM in both pretreatment methods. This means that AV did not decompose its matrix in simulated body conditions. On the other hand, AM decomposed its matrix well in the in vitro condition.

#### 3.5.3. Digestion and Absorption Efficiencies

**Figure 2:**
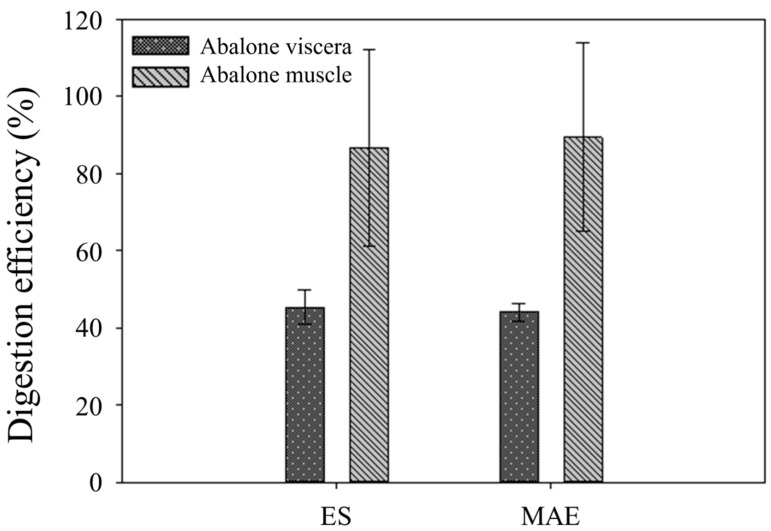
Digestion efficiency of iodine in abalone viscera and muscle after in vitro bioavailability test. ES and MAE mean European standard method and microwave-assisted extraction method, respectively.

**Figure 3:**
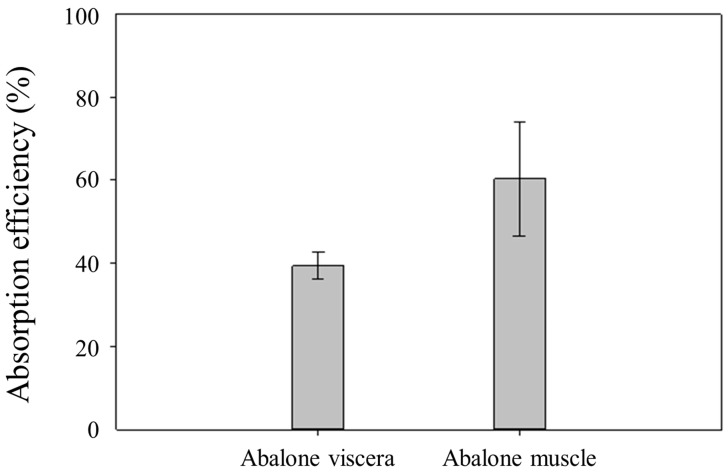
Absorption efficiency of iodine in abalone viscera and muscle after in vitro bioavailability test.

Digestion efficiency is defined as the sum of iodine in the inner membrane and outer membrane divided by the total iodine contents of the samples. This reflects the degree to which amounts of iodine in abalone samples (viscera or muscle) could be digested in simulated body conditions. The results are shown in Figure 2. In the case of AV, digestion efficiency showed 45 ± 3.6% for ES and 44 ± 1.9% for MAE. On the other hand, AM showed 87 ± 20% for ES and 89 ± 20% for MAE. These results mean that there were high possibilities that the AM matrix can be decomposed in our body condition easily. On the other hand, the AV matrix was not decomposed like AM, as the mass balance study suggested. There were no significant differences in the AV digestion efficiency between the two different pretreatment methods. Also, there were no significant differences in the digestion efficiency of AM between the two pretreatment methods. Figure 3 shows the absorption efficiency, which is defined as iodine in the inner membrane divided by iodine in the inner and outer membrane. This value explains the amount of iodine ingested after digestion compared to the total iodine in abalone. Through the dialysis membrane, 39 ± 2.6% and 60 ± 11% of iodine in AV and AM, respectively, were absorbed, indicating significant differences in absorption efficiency. The AV matrix can be decomposed within in vitro digestion conditions by approximately 45%, and the AM is able to release its iodine from the AM matrix by about 90%, as described in digestion efficiency. This means that compared with AV, AM can more effectively decompose their matrix in our bodies. Likewise, AM also has higher absorption efficiency than AV. According to WHO, our daily iodine recommended intake is 150 µg. This can be easily satisfied with just one abalone because one abalone weighs between 110 and 140 g, excluding its shell.

## 4. Conclusions

In this study, we determined the total iodine content in abalone (*Haliotis discus hannai*) and estimated its bioavailability using an in vitro method. The abalone was segmented into viscera and muscle sections. To measure the total iodine content, we utilized both the European standard method and a microwave-assisted extraction method. Given the limited number of previous studies quantifying total iodine in abalone, we conducted validations for these methods. The validation results, including linearity, limit of detection, limit of quantification, accuracy, and precision, confirmed that both the European standard and microwave-assisted extraction methods are suitable for determining the total iodine content in abalone. As a result, the total iodine content in abalone viscera exhibits levels of about 75.0–76.2 µg/g, while that in abalone muscle shows 16.2–17.8 µg/g. Additionally, we explored the in vitro bioavailability of iodine in abalone under simulated digestive conditions. The results show that abalone viscera exhibit levels of about 42.2% of digestion efficiency and 41.5% of absorption efficiency, while abalone muscle exhibit about 85.4% of digestion efficiency and 61.2% of absorption efficiency in simulated gastric and intestinal environments within a dialysis membrane.

## Figures and Tables

**Table 1:** Detailed instrumental information of ICP-MS.

ICP-MS Parameters	
Operating conditions	
RF power	1550 W
RF matching	1.8 V
Sample depth	8.0 mm
Carrier gas	
Argon (Ar)	1.1 L min^−1^
Helium (He)	4.3 mL min^−1^
Nebulizer pump	0.10 rps
S/C temp.	2.0 °C
Sample cone	Nickel, 1.0 mm orifice
Skimmer cone	Nickel, 0.75 mm orifice
Sample load time	
Uptake	30 s
Stabilize	40 s
Wash out	Ultrapure water for 40 s 1.0% TMAH for 40 s
Acquisition parameters	
Monitored signals	*m/z* 127 (^127^I), *m/z* 125 (^125^Te)
Integration time/mass	0.09 s (^127^I), 0.09 s (^125^Te)
Repetitions	3

**Table 2:** Linearity, limit of determination, accuracy, and precision test results.

Linearity	Range (µg/L)	Iodine Concentration Levels (0, 1, 5, 10, 20, 100)	
Limit of determination (*n* = 10)		ES ^(3)^	MAE ^(4)^
	LOD ^(1)^ (ng/g)	0.11	0.11
	LOQ ^(2)^ (ng/g)	-	5.4
Accuracy (*n* = 7)		ERM—BB422 (fish muscle)	
		ES	MAE
	Certified values (mg/kg)	1.4 ± 0.42	
	Found values (mg/kg)	1.5 ± 0.10	1.6 ± 0.066
Precision (*n* = 7)	Parameters	ERM—BB422 (fish muscle)	
		ES	MAE
	RSD_r_ (%)	6.7	4.0
	pRSD_R_ (%)	15.0	14.8
	HorRat	0.45	0.27

^(1)^ LOD: limit of detection. ^(2)^ LOQ: limit of quantification. ^(3)^ ES: European standard method. ^(4)^ MAE: microwave-assisted extraction method.

**Table 3:** Iodine determination in abalone viscera and muscle according to the pretreatment method.

**Pretreatment Method**	**Sample**	**Concentration (** **µ** **g/g)**
ES ^(1)^	AV ^(3)^	74 ± 2.2
	AM ^(4)^	17 ± 0.77
MAE ^(2)^	AV	76 ± 1.0
	AM	17 ± 0.51

^(1)^ ES: European standard method. ^(2)^ MAE: microwave-assisted extraction method. ^(3)^ AV: abalone viscera. ^(4)^ AM: abalone muscle.

**Table 4:** Recovery test results.

	Sample	Recovery (%)	
		ES ^(3)^	MAE ^(4)^
Low spike solution (5 µg/L)	Fish muscle (ERM—BB422)	102.4 ± 2.2	106.0 ± 1.8
	AV ^(1)^	99.2 ± 3.6	99.9 ± 3.0
	AM ^(2)^	104.0 ± 5.0	101.2 ± 5.7
High spike solution (10 µg/L)	Fish muscle (ERM—BB422)	94.5 ± 3.8	100.1 ± 1.9
	AV	98.3 ± 2.4	97.5 ± 2.3
	AM	101.2 ± 3.7	97.6 ± 3.5

^(1)^ AV: abalone viscera. ^(2)^ AM: abalone muscle. ^(3)^ ES: European standard method. ^(4)^ MAE: microwave-assisted extraction method.

**Table 5:** pH changes in samples during in vitro bioavailability test using a PIPES buffer.

Samples	Initial pH	Final pH	
		Dialysate pH	Residual Fraction pH
Method Blank	7.50	7.7 ± 0.09	8.0 ± 0.22
AV ^(1)^	7.50	7.2 ± 0.04	6.8 ± 0.09
AM ^(2)^	7.50	7.3 ± 0.03	6.9 ± 0.03

^(1)^ AV: abalone viscera. ^(2)^ AM: abalone muscle.

**Table 6:** Validation of in vitro bioavailability study.

Linearity		Dialysate	Residual Fraction
Limit of determination (mg/kg)	LOD ^(1)^	0.87	0.21
	LOQ ^(2)^	14	31

^(1)^ LOD: limit of detection. ^(2)^ LOQ: limit of quantification.

**Table 7:** Mass balance study for iodine (µg/g) during in vitro bioavailability test.

	Dialysate	Residual Fraction	Sum	Total Concentration	
				ES ^(3)^	MAE ^(4)^
AV ^(1)^	13 ± 1.5	20.4 ± 0.68	34 ± 1.7	74 ± 2.2	76 ± 1.0
AM ^(2)^	9.1 ± 3.4	5.6 ± 1.2	15 ± 2.9	17 ± 0.77	17 ± 0.51

^(1)^ AV: abalone viscera. ^(2)^ AM: abalone muscle. ^(3)^ ES: European standard method. ^(4)^ MAE: microwave-assisted extraction method.

## Data Availability

The original contributions presented in the study are included in the article, further inquiries can be directed to the corresponding author.
